# Supplementary material for: Experienced inclusion and recognition amongst people with spinal cord injury: A comparative study in Norway, The Netherlands, and Australia
Source: PLoS One. 2025 Apr 1;20(4):e0306231. doi: 10.1371/journal.pone.0306231 (PMC11960970; doi:10.1371/journal.pone.0306231)
Supplement: S1 Table — (DOCX) [file pone.0306231.s001.docx]

# Supporting information

## **S1 Table A**. Descriptive statistics over dependent variables

**S1 Table. Results on the questions on “social inclusion” and “people respect” in Norway, The Netherlands and Australia**

|  | Norway | | The Netherlands | | Australia | |
| --- | --- | --- | --- | --- | --- | --- |
| Social inclusion: | N | *%* | N | *%* | N | *%* |
| **Feel included when with others:** |  |  |  |  |  |  |
| *1, Not at all* | 4 | *0.6* | 10 | *4* | 61 | *4* |
| *2* | 27 | *4.5* | 20 | *8* | 138 | *9.1* |
| *3* | 83 | *13.9* | 57 | *22.8* | 353 | *23.2* |
| *4* | 191 | *32.1* | 79 | *31.7* | 483 | *31.8* |
| *5, Completely* | 290 | *48.7* | 83 | *33.3* | 483 | *31.8* |
| **Total** | 595 | *100* | 249 | *100* | 1,518 | *100* |
| Recognition/Respect |  |  |  |  |  |  |
| **People treat you with respect** |  |  |  |  |  |  |
| *1, Not at all* | 3 | *0.5* | 2 | *0.8* | 17 | *1.1* |
| *2* | 2 | *0.3* | 4 | *1.6* | 33 | *2.1* |
| *3* | 7 | *1.2* | 9 | *3.6* | 56 | *3.7* |
| *4* | 30 | *5.4* | 22 | *8.9* | 207 | *13.8* |
| *5* | 81 | *14.6* | 61 | *24.7* | 280 | *18.6* |
| *6* | 201 | *36.2* | 116 | *46.9* | 489 | *32.5* |
| *7, A great deal* | 230 | *41.5* | 33 | *13.3* | 423 | *28.1* |
| **Total** | 554 | *100* | 247 | *100* | 1,505 | *100* |
