# Supplementary material for: Experienced inclusion and recognition amongst people with spinal cord injury: A comparative study in Norway, The Netherlands, and Australia
Source: PLoS One. 2025 Apr 1;20(4):e0306231. doi: 10.1371/journal.pone.0306231 (PMC11960970; doi:10.1371/journal.pone.0306231)
Supplement: S2 Table — Controlling for country. (DOCX) [file pone.0306231.s002.docx]

## **S2 Table B**. Regression table for coefficient plot in figure 2

**S2 Table. Regression effect on perceived inclusion and recognition.** Controlling for country

|  | Inclusion | Inclusion | Recogn/Respect | Recogn/ Respect |
| --- | --- | --- | --- | --- |
| Completely dependent or use an electric wheelchair | - | - | - | - |
| Manual wheelchair | 0.013 (0.064) | -0.018 (0.062) | 0.014 (0.075) | -0.018 (0.072) |
| Walking with walking aids | 0.016 (0.068) | -0.016 (0.066) | 0.046 (0.080) | 0.015 (0.077) |
| Walking without walking aids | 0.171^*^ (0.069) | 0.054 (0.067) | -0.053 (0.081) | -0.204^**^ (0.078) |
| Male | - | - | - | - |
| Female | 0.019 (0.050) | 0.041 (0.048) | 0.083 (0.059) | 0.113^*^ (0.056) |
| Age of Respondent | 0.006^***^ (0.002) | 0.002 (0.002) | 0.012^***^ (0.002) | 0.007^***^ (0.002) |
| Low education | - | - | - | - |
| Mid education | 0.050 (0.056) | 0.029 (0.054) | 0.094 (0.066) | 0.062 (0.063) |
| High education | 0.126^*^ (0.061) | 0.110 (0.059) | 0.247^***^ (0.072) | 0.218^**^ (0.069) |
| No paid work | - | - | - | - |
| Paid work | 0.307^***^ (0.053) | 0.280^***^ (0.052) | 0.228^***^ (0.063) | 0.204^***^ (0.060) |
| The Netherlands | 0.027 (0.074) | 0.014 (0.071) | -0.097 (0.086) | -0.115 (0.082) |
| Norway | 0.368^***^ (0.056) | 0.324^***^ (0.054) | 0.449^***^ (0.067) | 0.398^***^ (0.063) |
| Australia | - | - | - | - |
| No influence/applicable |  | - |  | - |
| Made life harder |  | -0.661^***^ (0.051) |  | -0.905^***^ (0.059) |
| Constant | 3.281^***^ (0.119) | 3.708^***^ (0.120) | 4.721^***^ (0.141) | 5.305^***^ (0.140) |
| Observations | 2222 | 2194 | 2171 | 2141 |
| Adjusted *R*^2^ | 0.056 | 0.124 | 0.053 | 0.148 |

Standard errors in parentheses. ^*^ *p* < 0.05, ^**^ *p* < 0.01, ^***^ *p* <0.00
