# Supplementary material for: Experienced inclusion and recognition amongst people with spinal cord injury: A comparative study in Norway, The Netherlands, and Australia
Source: PLoS One. 2025 Apr 1;20(4):e0306231. doi: 10.1371/journal.pone.0306231 (PMC11960970; doi:10.1371/journal.pone.0306231)
Supplement: S3 Table — (DOCX) [file pone.0306231.s003.docx]

## **S3 Table C**. Regression table for figure 3 and 4

**S3 Table. Regression table for the effect on perceived inclusion and recognition for Norway, The Netherlands, and Australia**

|  | Norway | | The Netherlands | | Australia | |
| --- | --- | --- | --- | --- | --- | --- |
|  | Inclusion | Recogn/Resp | Inclusion | Recogn/Resp | Inclusion | Recogn/Resp |
| Completely dependent or use an electric wheelchair | - | - | - | - | - | - |
| Manual wheelchair | 0.052 (0.145) | 0.053 (0.165) | 0.569^**^ (0.210) | 0.661^**^ (0.211) | -0.096 (0.074) | -0.115 (0.088) |
| Walking with walking aids | 0.103 (0.132) | 0.133 (0.152) | 0.450^*^ (0.211) | 0.379 (0.211) | -0.104 (0.085) | -0.055 (0.100) |
| Walking without walking aids | 0.167 (0.131) | -0.094 (0.149) | 0.649^**^ (0.219) | 0.612^**^ (0.222) | -0.063 (0.088) | -0.359^***^ (0.104) |
| Not paid work | - | - | - | - | - | - |
| Paid work | 0.204^*^ (0.087) | 0.118 (0.099) | 0.311 (0.159) | -0.072 (0.161) | 0.309^***^ (0.068) | 0.294^***^ (0.081) |
| Male | - | - | - | - | - | - |
| Female | 0.052 (0.079) | 0.158 (0.090) | 0.343^*^ (0.146) | 0.164 (0.147) | -0.025 (0.065) | 0.097 (0.077) |
| Age of Respondent | 0.002 (0.002) | 0.010^***^ (0.003) | 0.008 (0.006) | 0.006 (0.006) | 0.002 (0.002) | 0.006^*^ (0.003) |
| Low education | - | - | - | - | - | - |
| Mid education | -0.055 (0.114) | 0.118 (0.131) | -0.086 (0.179) | -0.066 (0.182) | 0.073 (0.067) | 0.060 (0.079) |
| High education | 0.156 (0.114) | 0.281^*^ (0.131) | 0.009 (0.158) | 0.414^**^ (0.158) | 0.097 (0.079) | 0.160 (0.093) |
| No influence/applicable | - | - | - | - | - | - |
| Made life harder | -0.586^***^ (0.097) | -0.715^***^ (0.113) | -0.674^***^ (0.153) | -0.804^***^ (0.153) | -0.694^***^ (0.064) | -0.988^***^ (0.076) |
| Constant | 3.958^***^ (0.216) | 5.378^***^ (0.248) | 2.871^***^ (0.422) | 4.729^***^ (0.422) | 3.821^***^ (0.156) | 5.446^***^ (0.184) |
| Observations | 558 | 521 | 242 | 239 | 1394 | 1381 |
| Adjusted *R*^2^ | 0.105 | 0.116 | 0.117 | 0.150 | 0.095 | 0.124 |

Standard errors in parentheses; ^*^ *p* < 0.05, ^**^ *p* < 0.01, ^***^ *p* < 0.00
